# Tumor heterogeneity assessment using single-cell RNA sequencing (scRNA-seq): applications in lung cancer for diagnosis and treatment

**DOI:** 10.3389/fimmu.2025.1693784

**Published:** 2026-01-06

**Authors:** Cecilia Bica, Oana Zanoaga, Laura Pop, Cristina Ciocan, Lajos Raduly, Andreea Nuțu, Ioana Berindan-Neagoe, Andreas Bender

**Affiliations:** 1 Department of Genomics, MEDFUTURE Institute for Biomedical Research, Iuliu Hatieganu University of Medicine and Pharmacy, Cluj-Napoca, Romania; 2 Doctoral School, Iuliu Hatieganu University of Medicine and Pharmacy, Cluj-Napoca, Romania; 3 Romanian Academy of Medical Sciences, Bucharest, Romania; 4 College of Medicine and Health Sciences, Khalifa University of Science and Technology, Abu Dhabi, United Arab Emirates; 5 Centre for Molecular Informatics, Department of Chemistry, University of Cambridge, Cambridge, United Kingdom; 6 STAR-UBB Institute, Babeş-Bolyai University, Cluj-Napoca, Romania

**Keywords:** lung cancer, molecular profile, single-cell RNA sequencing (scRNA-seq), tumor heterogeneity, tumor microenvironment (TME)

## Abstract

Recent progress in single-cell RNA sequencing has led to mechanistic and clinically actionable insight into genetic heterogeneity and tumor progression *via* transcriptome profiling at single-cell level, applied to identification of cell types, gene expression patterns, and signaling pathways involved in cancer development. In this work, we review the use of single-cell RNA sequencing (scRNA-seq) applications to gain insights into tumor molecular and cellular characteristics, such as cellular heterogeneity, rare cell populations, characteristic pathogenic cell populations, cells of the immune tumor microenvironment, and information regarding clonal evolution, none of which can be observed using bulk RNA-seq. We describe how this set of methods facilitates a better understanding of tumor heterogeneity, interactions between the tumor cells and the cells of the tumor microenvironment (TME), and can elucidate potential therapeutic targets. From the applied clinical perspective, we summarise the ability of scRNA-seq data to identify molecular indicators for diagnosis, outcome, and prediction of response to therapy. This is particularly relevant due to the low response rate to therapy of non-small cell lung cancer (NSCLC) and acquired resistance, including in immunotherapy.

## Introduction

Cancer contributes considerably to the global disease burden. According to the most recent statistics, reports, and forecasts, the annual number of global cancer deaths is increasing—a trend expected to continue for at least two decades (1–5). One reason for this situation is related to the rise in the incidence of cancer following lifestyle changes, e.g. a growing middle class in developing countries which consumes more meat, alcohol, and cigarettes, etc. (6), although a decrease in mortality *rates* in cancer is observed in some developed countries, as according to a study investigating the UK population (37% decline in men and 33% decline in women aged 35–69 years) (7). Hence, cancer remains of enormous medical relevance to society.

One of the main determinants of therapy resistance in cancer is tumor heterogeneity. Evidence suggests that tumor heterogeneity is not solely caused by genomic instability but by cooperation with other elements, such as clonal evolution and selection, to maintain and promote tumor development and progression (8), and hence understanding those aspects in an integrated manner is key for both diagnosis and treatment. The hallmarks of therapy resistance are either inherited through the genetic and epigenetic makeup of cancer cells or acquired following treatment. The acquired form results from therapeutic interventions that foster the evolution of treatment-resistant cellular subpopulations (9, 10). The evaluation of tumor heterogeneity is therefore critical, distinct in every patient regarding spatial distribution and temporal heterogeneity, with associated (different) effects on tumor progression, recurrence, and resistance to therapy (11, 12). In this respect, the complex tumor microenvironment (TME), characterized as a highly structured ecosystem of elements, such as immune and stromal cells, blood vessels, and the extracellular matrix plays a vital role in cancer cell survival, invasion, and metastasis (13). The dynamics within the tumor need to be understood *spatially* and *over time*, as the tumor configuration includes various cell types – cancer cells, endothelial cells, fibroblasts, and immune cells, which incorporate multiple distinct cell populations (14). Different cell populations possess distinct molecular signatures, which confer varying levels of treatment sensitivity and ultimately shape the overall response to therapy (8). Such insights into tumor heterogeneity and dynamics needed for disease understanding and to drive treatment are currently being facilitated by novel techniques to generate and analyze single-cell ‘-omics’ data.

According to the International Agency for Research on Cancer, lung cancer remains the number one cause of cancer-related deaths, with 9.7 million deaths in 2022 (15, 16). The current state of knowledge is that actionable genomic alterations encountered in non-small cell lung cancer (NSCLC) tumors are present mainly in lung adenocarcinoma tumors, as opposed to squamous cell tumors (17). Targeting these driver alterations using suitable targeted treatment options improves overall survival (18). Still, in terms of absolute numbers, in lung adenocarcinoma (LUAD) tumors, oncogenic driver mutations were observed in only 27% to 41% of tumors, with variations related to the histologic subtype (19). It was noted that although complex subclonal heterogeneity characterizes recurrent lung cancer, the dominant oncogenic driver that was clinically relevant at the time of diagnosis (and initial surgical treatment) often still exists at the time of recurrence, indicating their role in conferring sensitivity to targeted therapy (19). Still, the majority of NSCLC patients do not harbor (currently known) driver mutations/alterations, thus indicating both a lack of biological understanding and our inability to select suitable treatment options currently. In particular, driver mutations are rarely present (20) in squamous cell carcinoma, and higher intratumor heterogeneity was observed in these tumors compared to lung adenocarcinomas (21, 22). The majority (about 70%) of NSCLC patients diagnosed and undergoing surgical resection in the early stages will develop recurrent metastatic disease (23). Thus, NSCLC is currently poorly treated due to a lack of molecular testing on early-stage specimens, as treatment strategies and prognosis may differ within the same stage due to its broad classification (24), and we still need to understand both disease biology and actionable signals in said biology much better. In lung cancer, heterogeneity can occur not only as interpatient heterogeneity, which will result in different treatment behavior in clinic, but also intratumor heterogeneity, which arises not solely from mutations in multiple genes, but also in terms of cell populations with different phenotypic features (25, 26). Stratifying NSCLC patients based on their molecular characteristics has improved with the development of single-cell sequencing technologies as our knowledge of the mechanisms underlying lung cancer has deepened. A high-resolution view of the cells in the TME in early and advanced stages of LUAD and offering insights regarding cellular and molecular network dynamics during tumor progression possible using single-cell sequencing technologies provides the fundamentals for future discoveries of molecular therapeutic targets (27). Moreover, the mechanism of resistance sensitivity to therapy can be identified by identifying rare cell populations/subpopulations and their role in regulating crucial biological pathways and patterns related to immune cell infiltration in lung tumors (28, 29). Considering the highly heterogeneous nature of NSCLC, identifying driver gene alterations specific to each lung cancer subtype can be done by adequately exploiting data generated using scRNA-seq (22, 30).

In addition to heterogeneity, practical reasons make tumor characterization in the clinical context difficult. According to the National Comprehensive Cancer Network (NCCN) (31) (as well as in the authors’ experience), in recurrent NSCLC, it is often difficult to obtain a suitable tissue sample for molecular profiling due to small sample sizes or lack of access to the tumor location. This situation creates disadvantages in accessing important molecular information regarding the histological profile, biomarkers, and other resolutions for molecular testing (32).

Given the progress of the field, the purpose of this review is to describe the use of scRNA-seq in exploring tumor heterogeneity with a particular focus on lung cancer and immune cell infiltration, and covering both diagnosis and treatment response.

### From RNA sequencing to single-cell RNAseq - methods

Single-cell and spatial RNA sequencing are analytical methods of significant current interest used to gain insights into tumor molecular and cellular characteristics in complex tumor heterogeneity (33, 34). Although in the last two decades, bulk RNA-seq was extensively used to characterize cancer biology, it enabled the clinical translation of only a few gene panels into clinical practice. The reasons for this were partially intrinsic to the data obtained since low-resolution data cannot characterize heterogeneous tumor biology (35, 36). More precisely, rare cell populations, characteristic pathogenic cell populations, cells of the immune tumor microenvironment, and information regarding clonal evolution cannot be observed using bulk RNA-seq (37). However, single-cell RNA-seq (scRNA-seq) and spatial transcriptomics methods developed in recent years contribute in obtaining a finer-grained picture of tumor heterogeneity and better understanding of interactions between tumor cells and cells of the TME and to assist the identification of novel therapeutic targets (38, 39). The reader is referred to recent reviews for a more detailed overview of single-cell and spatial transcriptomics sequencing (40–43) technologies. Different scRNA-seq platforms were developed, and they use different strategies for transcriptome profiling, which translates in differences of sensitivity, capacity, and reproducibility. Several studies were conducted comparing the sequencing methods/platforms to indicate which one should be used considering the sample type or size (44, 45). In the table below, we summarized the major sequencing platforms used for scRNA-seq, highlighting the strengths and weaknesses (**Table 1**).

**Table 1:** Comparative table summarizing strengths/weaknesses of major scRNA-seq platforms.

Sequencing platform/reference	Type/typical use	Strengths	Weaknesses
Illumina (46, 47)	Short reads/High-throughput gene quantification	-High trhroughput and accuracy -low error rate -well validated	-limited isoform resolution due to short reads -
Pacific Biosciences (48)	Long reads/Isoform characterization and full-length transcript profiling	-extremely accurate long reads -direct RNA sequencing	-lower throughput -high cost per base
Oxford Nanopore (48)	Long reads/Full-length isoform detection	-ultra long reads -direct RNA sequencing -detections of base modifications -portable devices	- higher raw read error rate in homopolymer regions - low capture efficiency
DNBSeq (49)	Short reads/High-throughput gene quantification	-low costs -high throughput -low duplication rates	-library compatibility still expanding -high technical noise

### Software and data analysis

Progress in data generation required the development of computational tools to analyze scRNA-sequencing data, yet the continuous expansion of these tools makes the establishment of best-practice workflows difficult. Currently various analysis pipelines for scRNA-sequencing exist, the most commonly used being Seurat (50, 51), Scanpy (52), and Bioconductor (53). For a more detailed review, see (41).

**Figure 1:**
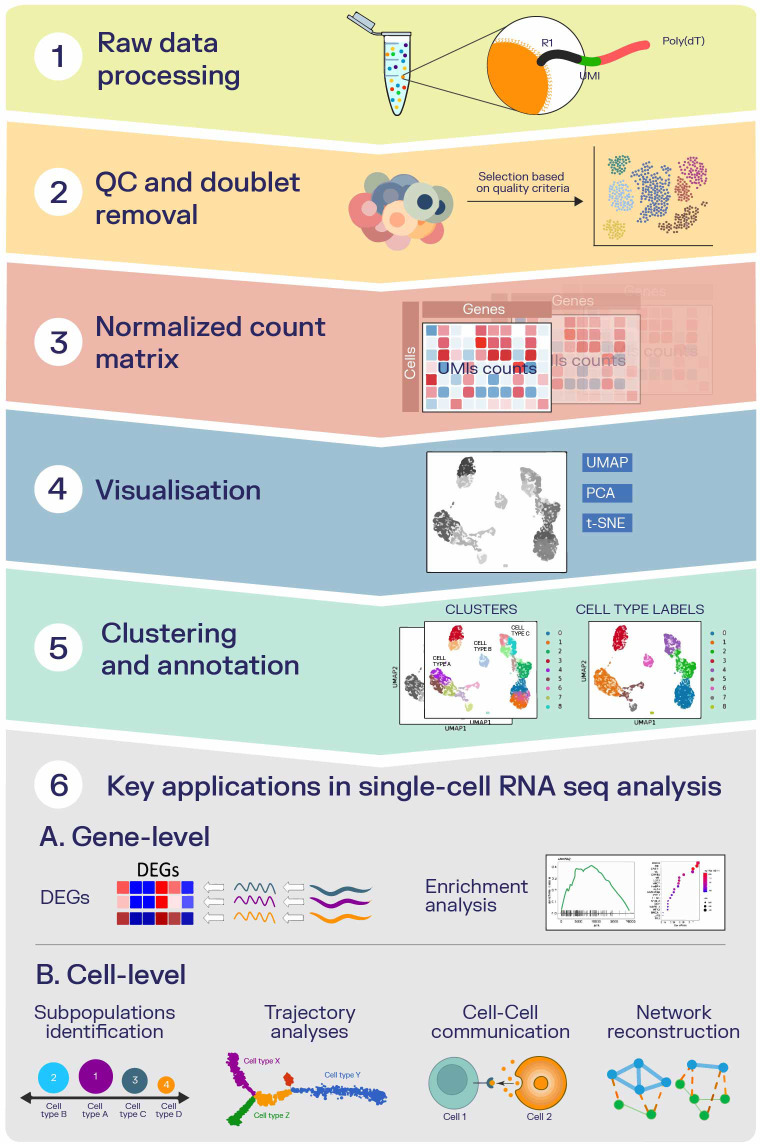
Analysis steps and applications using scRNA data which can generally be performed, with a focus in this review particularly on cancer-related applications. **1.** The initial steps require raw data processing - the mRNA sequence reads are mapped via cell barcodes or UMIs to the reference genome. **2.** Count matrices are processed with other filtering steps for QC (filter for doublets, low-quality, and dying cells. **3.** Data normalization - required to obtain accurate relative gene abundances between cells. **4**. Reduction techniques used in the manual investigation of the dataset related to clustering and cell type annotations – PCA, UMAP, and t-SNE. **5.** Clustering - the assignment of clusters is generally based on their typical classification markers. **6**. Exploratory analyses: **(A)** Gene-level - Identification of DEGs and selection of key genes that can represent biomarkers for cancer progression and enrichment analysis - aggregation of the entities in a DEGs list into pathways using GSEA. **(B)** Cellular-level – Subpopulations identification - to observe the abundance of different subpopulations, such as tumor, stromal, and immune cells, as well as rare subpopulations of cells; Trajectory analyses - enables the study of dynamic changes that are related to gene expression; Cell-cell interactions - influence multiple biological processes related to cancer, such as cellular growth and division, differentiation and progression; Network analysis – for understanding, predicting, or optimizing the structure and behavior of complex cancer systems.

The general scRNA data analysis workflow is illustrated in **Figure 1**. The initial steps require raw data processing (specific to the individual sequencing technology), including the mRNA sequence reads are mapped *via* cell barcodes or unique molecular identifiers (UMIs) to a reference genome (**Figure 1.1**). The resulting count matrices need to be processed with additional filtering steps for quality control, such as to filter for doublets, low-quality (e.g. damaged or stressed) and dying cells (**Figure 1.2**) (43, 54). Next, data normalization is required to overcome differences in gene expression counts that are generated by sampling effects of cells, to adjust systematic variations – the so-called “uninteresting” variation, which in scRNA-seq is generated by the effects of cell cycle on the transcriptome – and thereby to obtain accurate relative gene abundances between cells (**Figure 1.3**) (55). As the dimensions of the expression matrices are still high, visualization (**Figure 1.4**) requires reduction techniques, such as principal component analysis (PCA) (56), uniform manifold approximation and projection (UMAP) (57), and t-distributed stochastic neighbour embedding (t-SNE) (58). These techniques are often used in the manual investigation of the dataset related to clustering and cell type annotations (59). Clustering allows identifying immune cell populations and their abundance in experiments centered on solid cancer tissues. In studies centered on specific cell types in different cancer types using scRNA-seq, the assignment of clusters is generally based on their typical classification markers (**Figure 1.5**) (60), with more recent approaches being based on machine learning (e.g. deep learning) approaches (61); however, it needs to be kept in mind that in particular non-linear projection methods can be misleading and hence care needs to be taken in their interpretation (62, 63). Beyond visualization and cell type assignment, gene-level exploratory analyses (**Figure 1.6A**) can cover the expression data to identify differentially expressed genes, allowing the construction of regulatory networks and the establishment of a clonality tree (64). Identification of differentially expressed genes (DEGs) in cancer represents one key application in scRNA-seq analysis and it is used for detecting key genes that can represent biomarkers for cancer progression. When performing the analysis of DEGs in scRNA-seq, the biomarkers are detected for individual cell types, and hence on a much finer resolution, unlike in bulk RNA-seq where DEGs are identified in tumor vs. non-tumor/case vs. control/treated vs. untreated (65). The tools used to determine DEGs in scRNA-seq experiments are further outlined in a recent review (2019) (66), while another comprehensive review summarizes methods for gene selection using gene expression data (65). Gene set enrichment analysis (GSEA) is the most used tool for enrichment analysis. This tool aggregates the entities in a DEGs list into pathways (67).

In addition, intra-tumor heterogeneity can be explored in cellular-level applications (**Figure 1.6B**) to observe the abundance of different subpopulations, such as tumor, stromal, and immune cells, as well as rare subpopulations of cells, and explore their role in regulating cancer pathogenesis, angiogenesis and in mediating the immune response (68). DEG data can also provide input for various secondary analyses, such as the analysis of transcriptionally regulated pathways, gene sets or network analysis (69). Moreover, cell-cell interactions and information on the cell cycle can be explored using scRNA-seq data, as these interactions influence multiple biological processes related to cancer, such as cellular growth and division, differentiation and progression (70, 71). ScRNA-seq data can be used for cell trajectory analyses, as it enables the study of dynamic changes that are related to gene expression (34, 72). In the process of trajectory inference, genes that are associated with lineage of the trajectory can be identified, as well as genes that are differentially expressed between lineages (73) (**Table 2**).

**Table 2:** Types of analyses carried out using scRNA-seq data in cancer studies.

Analysis type	Example objectives	Example study/outcome
Subpopulation identification	Understanding the dynamics of cell evolutionary processes during disease progression and treatment – treatment-resistant cells Identification of cell populations with the ability to initiate new tumors and induce metastasis	-scRNA-seq data from untreated/chemotherapy treated lung cancer patients identified cell types (epithelial, endothelial, fibroblasts, and immune cells), and it was observed that the proportion of immune cells in tumor tissue was higher in treated patients than in the untreated ones, indicating that chemotherapy influences the architecture of TME and this may serve as a guide for making therapy more effective in these patients (74). - in small cell lung cancer (SCLC), a pro-metastatic subpopulation of cancer cells, characterized by high expression of *PLCG2* was identified, and patients in which this subpopulation represents more than 75% of the total cancer cells, had significantly worse OS compared to others (75)
Differentially expressed genes (DEGs)	Identification of key genes that can represent biomarkers for cancer progression	By comparing the DEGs in NSCLC subtypes, it was revealed that NK cells, Gran, and CD8 cells shared more common altered genes between LUAD and LUSC (76).
Cell-cell communication	Determination of intercellular communication pathways via aggregating ligand and receptor expression data for groups of cells to determine the groups of cells that are likely to interact with one another	Interaction between macrophages and neutrophils in the TME was observed in LUAD patients, indicating that TAMs communicate with neutrophils through the ICAM1 family, while neutrophils are recruiting TAMs via the CXCL family (77).
Trajectory analyses	Inferring the developmental or differentiation trajectories of cells Facilitating the reconstruction on evolutionary trajectories of different types of cell populations This assessment can be done spatially resolved (e.g. to understand the connection between different tumor sites or primary tumors vs. metastatic tumors) and temporally resolved (pre-treatment vs. post-treatment)	Single-cell transcriptomics data from head and neck tumors used to build trajectories of tumor-targeting T-cells from pre-nodal metastases in primary tumors using the Monocle 2.0 algorithm and discovered that these cells move in two different directions, the ‘naïve-dysfunction trajectory’ and the ‘memory- dysfunction trajectory’. This information can then be used for the identification of modulators for T-cell dysfunction in cancer (78, 79).
Network reconstruction	For revealing regulatory interactions between active transcription factors and target genes to provide insights for the identification of causal regulatory factors in biological processes	Possible immunotherapy options for bladder cancer patients were investigated using an interaction network which was constructed using 16 differentially expressed inflammatory genes, which indicated four altered pathways related to T-cell activation and leukocyte cell adhesion (80).
Enrichment analysis	Aggregates the entities in a DEGs list into pathways Scoring coordinated gene activity in cells in order to identify active pathways in each cell	After applying GSEA on different colorectal cancer patients RNA expression profile datasets, it was observed that in patients indexed as low-risk, based on a pyroptosis-related risk model, the pathways enriched were G2M checkpoint, IFα/γ response, DNA repair, and E2F targets; in high risk patients, the enriched pathways were hypoxia, coagulation, EMT, myogenesis, and downregulated response to UV (81).

EMT, epithelial to mesenchymal transition; IF, interferon; OS, Overall Survival; TAM, tumor associated macrophages; UV, ultraviolet.

## Applications of scRNA-seq in lung cancer

**Figure 2:**
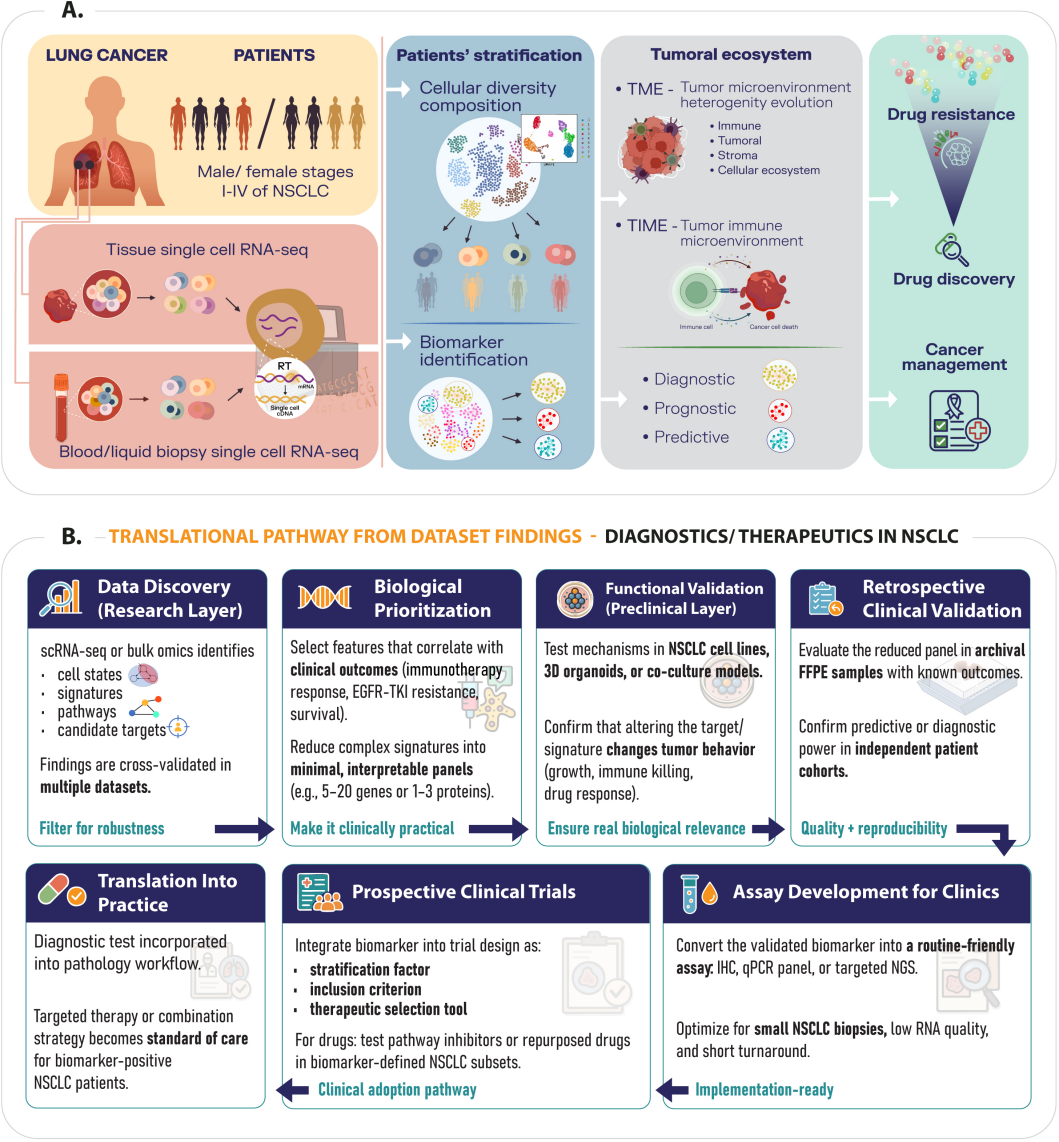
**(A)** Applications of scRNA-seq in translational research in lung cancer; **(B)** Translational pathway for operating dataset-derived discoveries into diagnostics and therapeutics for NSCLC.

In lung cancer research, scRNA-seq technology has been broadly used in applications such as biomarker discovery, the study of tumor heterogeneity, patient stratification based on the immune cell populations present within tumors, and in target and drug discovery (**Figure 2A**). Experimental scRNA-seq discoveries can be translated into clinical practice, following translational research, which include biomarker discovery, validation of drug targets, and development of therapeutic models, which lead to clinical implementation of these discoveries in clinical trials (82) (**Figure 2B**). These applications are also summarized in **Table 2**, and they will be described in more detail in the sections that follow.

### Biomarker discovery using scRNA data in lung cancer

*Diagnosis:* Considering the poor response rate of lung cancer patients and frequent acquired resistance, also in immunotherapy, early diagnostic biomarkers are required to detect and confirm the existence of this condition and are used to improve the classification, optimization of treatment options, and survival of these patients. A set of six mitophagy-related genes with diagnostic potential in NSCLC was developed by Yu et al. (83). The AUC for this model was 0.925, respectively 0.966, indicating its high predictive accuracy for the occurrence of NSCLC, in two different datasets (83). Another study identified that their Tumor Immune Dysfunction and Exclusion (TIDE) risk model had an AUC of 0.688 for the diagnosis of LUAD patients (84) (**Table 3A**). The ongoing investigations indicate that due to the highly heterogeneous nature of lung tumors, combined biomarkers represent a more valuable diagnostic power than single markers, as already reported in other types of cancer, such as ovarian (85), leading to an increase in the detection rate (86).

**Table 3:** Studies investigating biomarkers for the diagnosis, prognosis, and prediction of response to therapy in lung cancer using ex-vivo scRNA-seq data.

Lung cancer type	Scoring model	Signature	Outcome
A. Diagnosis			
NSCLC (83)	SVM and RF algorithms	6 mitophagy-related genes (*SRC*, *UBB*, *PINK1*, *FUNDC1*, *MAP1LC3B*, and *CSNK2A1*)	-high expression of *SRC*, *FUNDC1*, and *CSNK2A1* and low expression of *UBB*, *PINK1*, and *MAP1LC3B* associated with increased infiltration of naive B cells, M0/M1 macrophages, NK cells, plasma cells, T helper cells, and regulatory T cells in TT
LUAD (84)	Risk model	8 CAFs marker genes (*TIMP1*, *TPM2*, *NR2F2*, *MFAP4*, *SOD3*, *CAV1*, *SERPINH1*, and *FMO2*)	-risk model (AUC = 0.688) for LUAD diagnosis was developed -a positive correlation between the selected CAFs and EMT and Wnt/β-catenin signaling was found
B. Prognosis			
NSCLC (92)	SVM prediction model	12 key genes (*MS4A1, CCL5, GZMB, HBA1, TYROBP, CD74, LTB, EEF1A1, RPLP1, RPS18, GADPH*, and *ACTB*)	-survival probability in high risk vs. low risk group decreased over time - HR = 1.85 (1.36-2.53), p= 9.766e-05
NSCLC (93)	NETosis risk model	18 NETosis-Related Genes (*ALDH2, ALOX5AP, CCT6A, CD69, CKAP4, DDIT4, DOCK4, ERO1A, FBP1, FKBP4, KRT8, LDHA, MS4A1, S100P, SEC14L4, SLC16A3, SNX30*, and *UBE2S*)	-in nine different NSCLC cohorts, significantly worse prognosis was observed in patients with high vs. low score patients. HR values were in 1.56-3.22 range, p ≤ 0.006 for all cohorts
NSCLC (101)	Risk model	5 CAF marker genes (*FBLIM1, LAMC2, NDN, SEMA3C*, and *SLC39A14*)	-worse OS in low-score vs. high-score patients - HR = 2.31(1.59–3.34), p<0.0001
LUAD (90)	Risk model	7 NK cell marker genes (*GCSAML, ACTG1, ACOT7, SELENOK, PEBP1, BIRC3, ACAP1*)	-in six independent cohorts, high-risk patients had inferior prognosis than the low-risk patients - pooled HR = 2.22(1.78-2.78), p<0.0001
LUAD (103)	Risk model	7 exosome-related genes (*CCL20*, *MAP3K8*, *SEC61G*, *SLC34A2*, *CD79A*, *BIRC3*, and *RBM39*)	-patients in high risk group had poorer OS, with AUC ranges from 0.722 to 0.827 for 1-year, 0.636-0.706 for 3-year, 0.606-0.746 for 5-year, 0.5-0.726 for 7-year, and 0.621-0.708 for 10-year in different datasets
LUAD (104)	LPRI	15 prognostic-associated regulons (*HDAC2, FOSL1, MAFK, KLF16, MAFF, XBP1, HLF, PRDM16, PPARG, WT1, DLX2, NPAS2, E2F7, HOXC9*, and *TEAD4*)	-patients with high LPRI had a lower OS probability, with AUC ranges from 0.7 to 0.96 for 1-year, 0.57–0.79 for 3-year, and 0.58–0.81 for 5-year in different datasets
LUAD (100)	Risk model	4 CAF-related genes (*FAP*, *FDGFRB*, *ACTA2*, and *NOTCH3*)	-AUCs of high risk group range from 0.64 to 0.69 for 1-year, 0.66-0.68 for 3-year, 0.64-0.84 for 5-year in different datasets.
LUAD (105)	Risk model	5 NK cell markers (*ADRB2, IDH2, SFTPC, CCDC69*, and *CCND2*)	-AUCs of high risk group were 0.703 and 0.658 for 1-year in two datasets.
LUAD (106)	Risk model	3 necroptotic anoikis-related genes (*KRT6A*, *HMMR*, and *FAM83A*)	-higher expression of genes correlated with lower OS rates; AUCs of high risk group were 0.720 for 1-year, 0.733 for 3-years, 0.669 for 5-years
LUAD (107)	Risk model	9 disulfidptosis associated genes (*TLE1, LDHA, SHC1, EMC6, HTATIP2, JAG1, EPHX1, MYO6*, and *HERPUD1*)	-AUCs of high risk groups ranges from 0.565 to 0.92 for 1-year, 0.65-0.77 for 3-year, 0.62-0.83 for 5-year in different datasets
LUAD (108)	Risk model	5 disulfidptosis associated genes (*ERO1A, KRT18, GALNT2, PPIA*, and *CAPN12*)	-AUCs of high risk group were 0.77 and 0.65 for 1-year, 0.73 and 0.68 for 3-year, 0.65 and 0.66 for 5-year in two different datasets
LUAD (109)	Risk model	8 cuproptosis-related stemness genes (*KLF4, SCGB3A1, COL1A1, SPP1, C4BPA, TSPAN7, CAV2*, and *CTHRC1*)	-AUCs of high risk group were 0.704 for 1-year, 0.704 for 3-years, 0.683 for 5-years
LUAD (110)	Risk model	16 ATNKGS (*CD28, CTSL, HLA-DOB, HSPA2, HSPA6, IL2, MAP2K1, NCR3, NRAS, PAK2, PTPN6, RAET1E, RFX5, RFXAP, SHC1*, and *TAP2)*	-patients in high risk group had poorer OS outcomes in six different datasets – pooled HR = 2.55 (2.05–3.17), p<0.0001
LUAD (111)	Risk model	4 muscle failure-related genes (*BDNF, FNDC5, IL15*, and *MSTN)*	-in two different cohorts, this model proved to be a prognostic marker for OS in LUAD patients: HR=4.383 (3.056–6.285), p<0.0001 HR=1.337 (1.142–1.566), p<0.0001
LUAD (112)	Risk model	12 angiogenesis-related genes (*HPGD, IRX2, SFTPB, CHIA, HOXD1, HSD17B6, MUC16, S100P, C1orf116, KRT16, EGLN3*, and *ALC2A1*)	-AUCs of high risk group were 0.71, 0.71, 0.68 and 0.70 for 3-years; HR = 3.12(2.36–4.12), p<0.001
LUAD (84)	Risk model	8 CAFs marker genes (*TIMP1*, *TPM2*, *NR2F2*, *MFAP4*, *SOD3*, *CAV1*, *SERPINH1*, and *FMO2*)	-AUCs of high risk group range from 0.649 to 0.876 for 1-year, 0.572-0.709 for 3-year, 0.584-0.638 for 5-year in different datasets
LUAD (113)	Adenosine-related prognostic signature	3 adenosine-signaling-related genes (*CD4, FGR*, and *MNDA*)	-the signature is an independent prognostic indicator of OS in LUAD - HR = 3.032(1.339–6.865), p=0.008
LUSC (91)	TCMGrisk	5 T-cell marker genes (*BTG1, JUND, IER3, ZNF331* and *PSAP*)	-AUCs of high risk group range from 0.669 and 0.661 for 1-year, 0.603 and 0.628 for 3-years, 0.645 and 0.590 for 5-years in two different datasets
LUSC (114)	Risk model	4 apoptosis-related genes (*BMP2*, *GPX3*, *JUN*, and *AIFM3*)	-AUCs of high risk group were 0.682 for 3-years, 0.652 for 5-years, and 0.679 for 7-years
C. Response Prediction			
NSCLC (101)	Risk model	5 CAF marker genes (*FBLIM1, LAMC2, NDN, SEMA3C*, and *SLC39A14*)	-high risk patients had poor response (14%) to immunotherapy compared to low-risk patients (45%)
LUAD (90)	Risk model	7 NK cell marker genes (*GCSAML, ACTG1, ACOT7, SELENOK, PEBP1, BIRC3, ACAP1*)	-low risk group associated with increased response to PD-L1 therapy -risk score models could predict anti-PD-L1 response with 76.1% accuracy.
LUAD (100)	Risk model	4 CAF-related genes (*FAP*, *FDGFRB*, *ACTA2*, and *NOTCH3*)	-in low-risk group, the proportion of stable disease/progressive disease patients was lower (0.31/0.64) than that in high-risk group (0.15/0.38) in two different datasets
LUAD (98)	CRD score model	23 circadian-related genes (*ADA, C1QTNF7*, *CACNA2D2, CELF2, CLOCK, DIXDC1, EPHX1, FAT1, GIMAP5, HERPUD1, ICOS, KCNMA1, MICU3, NAALAD2, NAP1L5, NRXN1, OPHN1, PBXIP1, RBM38, RPS6KA5, SEC61G, SFTPC*, and *SLC25A42*)	-patients in high score group had higher response rate to TKIs, with AUCs of 0.927, 0.882, 0.829, 0.782, 0.836, and 0.853 between PD/NR, and RD patients
LUAD (99)	Gln risk model	10 Gln metabolism related genes (*EPHB2, CAV2, RTN2, SCPEP1, UNC5D, FURIN, PITPNC1, CH25H, RGS20*, and *TSPAN11*)	-low risk group associated with higher response rates (95%) to anti-PD1 and anti-CTLA4 immunotherapy compared to those in high risk group (88%)
LUAD (94)	Expression level	*SLC7A11*	-patients with low *SLC7A11* expression levels were more likely to benefit from immunotherapy
LUAD (113)	Adenosine-related prognostic signature	3 adenosine signaling–related genes (*CD4, FGR*, and *MNDA*)	-patients with high adenosine-related prognostic score had a poor PFS after immunotherapy

AT2, type II alveolar cells; ATNKGS, APC/T/NK cells-related gene signature; CAF, cancer-associated fibroblasts; CRD, circadian rhythm disruption; Gln, glutamine; ICD, immunogenic cell death; LPRI, Lung Cancer Prognostic Regulon Index; NETosis, Neutrophil Extracellular Traps process; NK, natural killer; OS, overall survival; PD/NR, progressive disease/nonresponse; PFS, progression-free survival; RD, response; TCMGrisk, T-cell marker genes risk score; TKIs, tyrosine kinase inhibitors; TT, tumor tissue.

*Prognostic biomarkers:* Prognostic biomarkers aim to estimate the likelihood of a future clinical event of patients and they usually accompany clinical markers, such as TNM stage, histologic subtype, lymph node involvement, and the presence of metastases (87). Overall, predictive biomarkers for targeted therapy (e.g., EGFR, ALK) and immunotherapy (e.g., PD-1/PD-L1) have improved outcomes for many NSCLC patients. However, new therapeutic predictors are still needed to ensure better outcomes, particularly for those with the LUSC histotype (88). For LUAD and LUSC tissues, by combining scRNA-seq data with deconvoluted bulk RNA-seq data, Zhang et al. (89) intended to identify cellular subtypes for each lung cancer histotype. They were able to observe using the log-rank four main groups based on cellular composition for each histotypes, and concluded that increased percentages of type II alveolar cells (p=0.0043) and basal cells (p=0.0038) in LUSC, and increased percentages of fibroblasts (p=0.016) in LUAD predicted poor survival in these patients, indicating the contribution of cellular composition to assessing the prognosis of lung cancer patients (89). A conceptually similar approach was used by Song et al. (90) to uncover a prognostic gene signature based on natural killer cells markers in LUAD patients. A specific seven-gene prognostic signature expressed in various cell clusters was established in this study. When patients were categorized in low-/high-risk based on the median risk score to assess survival status and the prognostic signature was validated in clinical subgroups based on age, smoking status, gender, stage indicating its predictive stability (HR: 2.227, 95% CI: 1.782–2.784, p<0.0001) (90). To emphasize the importance of T-cell marker genes in LUSC patients, a prognostic signature consisting of five T-cell marker genes was developed in a different study and validated on two different cohorts (91). Given those relatively low AUC values it can be seen that the model doesn’t capture all biological complexity in LUSC at this stage. In another study a supervised machine algorithm was used to identify 12 key genes in a prediction model using scRNA-seq data available in Gene Expression Omnibus (GEO) with prognostic potential in NSCLC. By applying Cox-proportional hazards regression model on two different NSCLC cohorts it was observed that survival probability in low risk vs. high risk group decreased over time (HR = 1.85 (1.36-2.53), p <0.0001) (92). Another risk score calculated based on Neutrophil Extracellular Traps process (NETosis) related genes in LUAD scRNA-seq data was shown to be rather predictive in assessing NSCLC patient survival. Using nine different NSCLC cohorts, significantly worse prognosis was observed in patients with high vs. low score patients (Hazard Ratios (HRs) values fall within 1.56-3.22 range, p < 0.01 for all 9 cohorts) (93). Also solute carriers seem to be suitable as prognostic markers, the role of *SLC7A11* as an independent prognostic indicator in LUAD patients was revealed using scRNA-seq data combined with bulk RNA-seq data (94). It was observed that elevated expression of this gene was observed in clusters that were labeled as epithelial cells, and increased levels of *SLC7A11* indicated poor OS for these patients (94), likely due to differences in oxidative stress response (95). As illustrated in **Table 3B**, using scRNA-seq data, genes/markers of prognosis can be distinguished for specific pathways or markers of the immune microenvironment encountered altered in cancer. Most of the studies evaluated the stability of these markers in predicting the outcome of NSCLC patients for 1-, 3-, and 5-years. It can be noted that often relatively similar Hazard Ratios are obtained in studies such as the above, which is likely due to the underlying correlation structure and relatively low dimensionality of biological readout space, which often gives relatively similar predictive value, largely independent of the variables employed in models (96). Hence, the understanding and prediction of prognostic biomarkers still require significant future progress in both research and clinical translation.

*Drug Response Prediction:* Predictive biomarkers are used to predict the likelihood of a patient to respond to a specific treatment, as well as in stratification of these patients based of their probability of response (97). The risk score calculated based on the gene signature for prediction of natural killer cell markers developed by Song et al. (90) for LUAD was valuable for predicting response to PD-L1 therapy with 76.1% accuracy (90). Another study that focused on genes enriched in circadian-related signaling pathways using scRNA-seq data of LUAD patients established a ‘disruption score’ to assess the response of patients to tyrosine kinase inhibitors (TKIs) (98). Also in the immunotherapy area a study analyzing glutamine metabolism in LUAD, a risk model including 10 genes was constructed that divided patients into low-risk and high-risk groups. Based on this score, it was observed that not only in LUAD patients, but also in other types of cancer (melanoma and urothelial cancer), the low risk group was associated with lower non-response rates (of 5%) to anti-PD1 and anti-CTLA4 immunotherapy compared to those in the high risk group (where the value was 12%) (99). Using a risk score based on a signature consisting of 4 CAF-related genes, Ren et al. (2023) demonstrated that low-risk patients were more likely to have a partial or complete response to PD-L1 blockade immunotherapy, as the proportion of stable disease/progressive disease patients was lower (0.31/0.64) in the high-risk groups compared to low-risk groups (0.15/0.38) in two different datasets (100). Similarly, another risk score calculated using the TIDE algorithm including a set of five CAF marker genes indicated that high-score patients had reduced CD8 and elevated CAF-markers expression, indicating these patients had a poor response to immunotherapy (101) (**Table 3C**). Resistance to immunotherapy was investigated in a study in which NSCLC patients were categorized as those who experienced major pathologic response and those with non-major pathologic response. Differences among the two groups were observed in cell populations and highly expressed ACTN4, ATF3, BRD2, CDKN1A, and CHMP4B in epithelial cells of non-major response pathologic response patients represent potential biomarkers associated with resistance to immunotherapy in NSCLC (102). All these signatures indicate a large number of molecular indicators for diagnosis, outcome, and prediction of response to therapy, which need further exploration and validation in a clinical setting.

### Tumor heterogeneity characterization of the TME using scRNA-seq

Tumor heterogeneity, a major challenge in oncology and a primary cause of drug resistance (115), arises from differences among cancer cells in their transcriptional profiles, morphology, and metabolism. These variations occur both within individual tumors and between different tumors. In addition, recent data indicated that tumor heterogeneity also includes characterization of the TME, as well as cell-cell interactions and their dynamics as these factors significantly influence treatment efficiency in cancer patients (116). Drug resistance originating from tumor heterogeneity is encountered in all cancer types and it covers all therapeutic modes – chemotherapy, radiotherapy, immunotherapy, and targeted therapy (117), thus highlighting the relevance of characterizing the temporal and spatial evolution in tumor development and progression to unravel mechanisms of drug resistance. Using scRNA-seq enables heterogeneity assessment in tumor and adjacent non-tumor tissue and peripheral blood, to reveal knowledge regarding new cell populations with characteristic gene expression profiles, cell-cell communication, cellular differentiation, and gene regulation networks in the TME (118–120). The high resolution of individual cells assisted by scRNA-seq allows for an improved understanding of both intra- and inter-tumoral heterogeneity (121), where the characterization of the tumor-infiltrating immune cells are able to assist in predicting responsiveness to immunotherapies. The characterization of the tumor immune microenvironment (TIME) can be done considering the different populations and total number of immune cells present within a tumor, but also considering the spatial distribution of these cells to understand aspects related to recruitment and activation of immune cells, as means to develop new immunotherapies (38). Considering the observations indicating that sex-related differences in the prognosis of NSCLC patients are primarily due to immune response variation (122), a recent investigation (123) evaluated differences in cellular compositions and gene expression profiles in immune cells of the TME among men and women with NSCLC. The dissimilarities identified were in DEGs in tumor associated macrophages (TAMs), where *C1QC*, a complement-related gene is highly expressed in female-derived TAMs and associated with poor prognosis, while *FN1* and *SPP1* were highly expressed in male-derived TAMs. This suggests that male patients with higher expression of *SPP1* in TAMs are more likely to benefit from adjuvant immunotherapy, as this gene was reported as potential target for this type of treatment, improving its efficacy (123). When analyzing NSCLC subtypes, it was observed (76) that CD8, NK, and Gran cells had a common pattern of altered genes in the two main subtypes, LUAD and LUSC, while dendritic cells, T-regs, CD4 cells, and macrophages had fewer overlapping DEGs. A more detailed analysis of the macrophages showed that these cells had more immune functions, such as shaping the TME, in the progression in LUSC than in LUAD, with different dominant subclusters for the two subtypes, namely *FABP4*-macrophages in LUAD and *SPP1*-macrophages in LUSC, the latter being reported as promoting to lung fibrosis. This evaluation indicates significant differences among LUAD and LUSC TIME, which can potentially be exploited for therapy (76, 124, 125). Another recent study indicated that analyzing NSCLC patients that were treated with chemotherapy combined with neoadjuvant immunotherapy based on TIME features can indicate patterns related to response to therapy, as the expansion of different immune cell types expresses the ability to target malignant cells. An immune signature of exhausted T cells was determined that was efficient in identifying patients at risk of recurrence that were categorized as non-major response (126). Although SCLC has long been considered a relatively homogeneous neuroendocrine carcinoma, recent single-cell RNA sequencing studies have demonstrated extensive intratumoral heterogeneity and striking lineage plasticity (75, 127–129). Tumor heterogeneity in SCLC early-stage tumors was investigated and it was observed that malignant cells had DEGs, such as downregulated genes involved in the immune response - *CD74*, *IDO1*, and *ISG15* – highlighting the existing (destructive) synergy between the immune and tumor cells (130). Moreover, the intertumor heterogeneity of SCLC tumors was reflected in differentially expressed transcription factors specific for SCLC subtypes - *ASCL1*, *NEUROD1*, and *POU2F3* (130). DEGs from circulating tumor cell (CTC) and CTC-derived xenografts from SCLC patients using single-cell profiling, indicated variable expression of *DLL3*, which is an inhibitory NOTCH ligand, a therapeutic target for which several targeted therapies are currently in clinical trials (chimeric antigen receptor (CAR) T cell therapy and CAR-NK). On the other hand, rovalpituzumab tesirine - an antibody-drug conjugate targeting *DLL3* was recently discontinued as it failed as a third-line treatment for SCLC-, as *DLL3* it was reported as having a higher expression level in SCLC, indicating limited efficacy on its target (131, 132). This implies that expression heterogeneity of *DLL3* may contribute to therapy resistance, as its expression was dynamic and in some cases faded after chemotherapy, suggesting a potential mechanism for adaptive resistance in targeted therapy and the importance of timing in administration of targeted therapy. Analyzing CTC collected at different time points of treatment advance, the lowest amount of CTCs was present in samples during treatment response, while samples collected after relapse had the highest number of CTCs (132). These findings and their relevance according to different lung cancer histotypes are synthesized in **Table 4**, considering the contribution of scRNA-seq analyses in characterizing tumor cells, lineage and evolution, immune miroenvironment, stromal cells, and therapeutic implications (**Table 4**). In SCLC, further scRNA-seq investigations are needed to offer a broad image of intratumoral heterogeneity and its implications in resistance to immunotherapy, although the current limitations are widely acknowledged – rare primary human SCLC tumor samples (129), longitudinal data remain scarce (128), and the tumor immune landscape is incompletely characterized (75). Assessing the immune environment of tumors and identifying different subclasses based on their immune landscape has hence overall become essential for further developments in immunotherapy, where scRNA-seq provides the data for resolving mechanisms related to immune-modulating therapies.

**Table 4:** Relevance of scRNA-seq findings across lung cancer subtypes.

Category	LUAD	LUSC	SCLC
Tumor cell characterization	Identified multiple transcriptional tumor cell states (AT2-like, EMT-like, hypoxic, proliferative) that are not visible by bulk RNA or histology (133, 134).	Revealed squamous lineage states (basal vs differentiating) and stress-response programs, showing greater cellular heterogeneity than expected from morphology (89).	Discovered coexistence of neuroendocrine and non-neuroendocrine tumor states and dynamic switching between them, especially after treatment (128).
Lineage and evolution	Pseudotime analysis confirmed AT2 cell origin and mapped progression from pre-malignant to invasive states (133).	scRNA-seq traced lineage to airway basal stem cells and showed limited lineage plasticity compared to LUAD (89).	scRNA-seq demonstrated subtype plasticity over time, defining “state switching” as a mechanism of treatment resistance (75).
Immune microenvironment	Showed immune-inflamed TME with exhausted CD8^+^ T cells, plasma cells, and diverse macrophage populations not detected in bulk profiles (135).	Revealed that T cells are present but spatially excluded and suppressed by neutrophils and macrophages—information missed by single-cell–free platforms (136).	Showed immune desert features with almost complete absence of effector T cells and dominance of suppressive stromal/myeloid cells (130).
Stromal insights	Identified distinct CAF subtypes (inflammatory CAFs, ECM-rich CAFs) with defined signaling to immune cells (137).	scRNA-seq mapped dense CAF–immune interactions, especially TGF-β and matrix programs driving immune exclusion (125).	Identified stromal signaling pathways that support neuroendocrine-to-non-neuroendocrine transitions during relapse (128).
Therapeutic implications	Mapping of resistant subclones (e.g., EGFR-TKI–persistent cells) and immune phenotypes linked to immunotherapy response (98).	Showed stromal/myeloid pathways (e.g., TGF-β, CD36) as potential therapeutic targets rather than focusing only on checkpoint blockade (136).	Supported therapeutic strategies targeting cell-state transitions (e.g., NOTCH, MYC, YAP) rather than only immune-based treatment (132).

AT2, type II alveolar cells; CAF, cancer-associated fibroblasts; ECM, extracelluar matrix.

### Patient stratification based on T(I)ME architecture using scRNA-seq

Patient stratification can significantly increase the likelihood of clinical success (138) and hence there is hope that scRNA-seq data provides further opportunities in the future. This involves, in particular, the dynamics between immune and cancer cells, which enables us to explore the mechanisms underlying resistance to immunotherapy with TME involvement.

The majority of scRNA-seq studies that explored the TME in lung tumors were focused on characterizing T cell populations, as recent observations indicate that resistance to immune checkpoint inhibitors is associated with exhausted T cells phenotype (139). In this regard, in one study (140) that sequenced T-cells isolated from tumor and normal adjacent tissues and peripheral blood from NSCLC patients, the authors ascertained that based on the patterns of TILs patients could be divided into two groups, one enriched for pre-exhausted CD8+ T cells, non-activated T-regs and activated CD4+ cells, and the other one enriched for exhausted T cells and activated T-regs, with patients in the first group showing an improved outcome in terms of OS (140). Using both tumor tissue and adjacent non-tumor tissues from 7 untreated early-stage LUAD patients, the same clusters of T cells were found in both tumor and non-tumor tissues. Yet, a more detailed analysis revealed T cells in tumor tissues highly expressed exhaustion and regulatory markers, such as *TIGIT, LAYN, FOXP3*, and *CTLA4*. T cells from non-tumor tissues had a higher expression of markers for naïve and effector T-cell markers, indicating an immunosuppressive TME in early-stage LUAD which was characterized by T cells that differentiate into exhausted and regulatory subtypes, indicating the importance of the TME profile and tumor-TME interactions for future drug discoveries in LUAD (141). In NSCLC, exploration of tumor tissues before and after immunotherapy combined with chemotherapy was investigated using scRNA-seq from 3 pre-treatment and 12 post-treatment patients. Analysis on how the TME of the immune system and cancer cells dynamics change in response to neoadjuvant PD-1 blockade combined with chemotherapy was performed. It was observed that in patients with major pathologic response, defined as less than 10% residual viable tumor cells present in HE (haematoxilin and eosin) staining following treatment (142), MHC-II genes were highly expressed, indicating that the MHC-II pathway is a possible mechanism used by the TME to boost response to immunotherapy. Moreover, the tumor microenvironment (TME) differed between patients with distinct pathologic responses to immunotherapy, distinguishing good from poor responders. In good responders, cytotoxic T-cells were activated and recruited, while immunosuppressive cells—such as T-regs, CCL3+ neutrophils, and SPP1+ TAMs—were less abundant. In contrast, poor responders showed cytotoxic cell activation only at the onset of therapy. In contrast, the immunosuppressive cells were more abundant in the TME. Hence, these observations suggest that different pathological responses in patients are reflected in significant differences in the TME remodeling following therapy (143). Another stratification of patients considering the cellular composition of the TME was performed by Bischoff et al. (2021) (135) in LUAD samples. The patients/tumors were divided into (1) those with normal-like myofibroblasts, conventional T cells, NK cells, non-inflammatory monocyte-derived macrophages, and myeloid dendritic cells in one group, and (2) those characterized by cancer-associated myofibroblasts, exhausted CD8+ T cells, proinflammatory monocyte-derived macrophages, and plasmacytoid dendritic cells into the other group. Based on this signature, the first group was characterized as possessing a TME inert pattern, while the latter showed a TME activated pattern. However, this separation did not precisely correspond to the separation based on histological grades of LUAD tumors, although a signature of 20 genes based on this TME pattern indicated that patients with inert TME pattern had a better OS than those with activated TME pattern (HR = 0.5, 95% CI 0.35-0.71, p = 0.0001). These findings indicate that characterizing the TME at single-cell level represents a potential tool in assisting therapeutical decision making (135).

### Recent advances in drug and target discovery in lung cancer using scRNA-seq

Drug discovery in cancer remains difficult and expensive, not least due to an insufficient understanding of tumor biology, disease-related mechanisms, heterogeneity in disease response, and identification of efficient actionable therapeutic targets (82). One approach to match patient transcriptomics to compound treatment has been facilitated by the development of Connectivity Map (CMap) which, combined with integration of scRNA-seq data and cancer cell lines drug response analyses from the Library of Integrated Network-based Cellular Signatures (LINCS) has shown ways how to use cellular gene expression data for drug repurposing and target discovery’ of successful drugs able to target specific cell subpopulations (144, 145). However, the data in Cmap has been derived from cell culture systems, hence while it considers different cell types, it does not represent primary tumors, and also not tumor heterogeneity in the assay system used.

This section compiles recent examples of therapeutic agents and targets explored in lung cancer in *in vitro* and *in vivo* studies, which are listed in **Table 5**.

**Table 5:** Therapeutic effect investigated using scRNA-seq in different subtypes of lung cancer.

Drug/target	Cancer type	Study type/model	Effects	References
Therapeutic agents				
AKR1B1 knockdown using siRNA	NSCLC	*In vitro* - Human H2009 and SW480 cell lines Translational/tissue samples	Inhibition of cell proliferation Stimulation of cell apoptosis Identification of a subpopulation of mixed-lineage tumor cells	(146)
Vandetanib	NSCLC	*In vitro -* LC2/ad, LC2/ad (rep), LC2/ad-RPC-9 and VMRC-LCD cell lines	Diverse expression patterns of individual cells	(147)
Erlotinib	LUAD	PDX model	Drug-tolerant persisters (DTPs) induction	(148)
SELENOP-Mφ	NSCLC	Translational/40 samples of tumor and normal tissue	Inhibition of tumor progression	(76)
Therapeutic targets				
PTPRC, CCR2, SLAMF1, and HLA-DQA2	LUAD	Data from TCGA-LUAD cohort	Prediction of outcome and immune landscape	(149)
CBFA2T3, CR2, SEL1L3, TM6SF1, TSPAN32, ITGA6, MAPK11, RASA3, and TLR6	LUAD	Data from TCGA-LUAD cohort	Outcome prediction	(150)
VEGFA, TIMP1, and SPP1	LUAD	Data from GEO database	Association with poor prognosis	(151)
CD36	LUSC	Data from GEO Database	Regulation of immune and energy metabolism signals	(136)

After assessing the transcriptomic heterogeneity of several LUAD cell lines, gene expression alterations in response to vandetanib were evaluated. It was found that even though vandetanib directly targets EGFR and RET, no changes in their expression patterns were observed after treatment. Thus, more in-depth determinations are needed to underlie the mechanisms of acquiring drug resistance in these cells, such as transcriptomic variances between individual cells using more cell types and different environmental conditions (147).

One significant difficulty in achieving better outcomes in patients with *EGFR-*mutant lung cancer is related to the persistence of drug-tolerant cancer cells. Therefore, to investigate the role of different cell populations in tumor recurrence and to trace the origins of these drug-tolerant persistent cells, tyrosine kinase inhibitor treatment in the EGFR-mutant lung cancer PDX model was applied in one study (148) which inhibited tumor growth and induced drug-tolerant persistent cells. Moreover, the treatment significantly stimulated these pathways and produced alterations in the CAF population. All these observations indicate that NF-κB and IL-6/JAK/STAT3 pathways represent potential targets to be further investigated to overcome recurrence in these patients (148).

In a study which aimed to evaluate the immune landscape of NSCLC to underline its importance in developing efficient immunotherapy approaches and for the prediction of clinical responses, scRNA-seq on 72,475 immune cells from 19 NSCLC patients was employed to determine their immune cell transcriptome atlas (76). After outlining the immune heterogeneity of LUAD and LUSC in terms of Mφ and lymphocytes, a novel lymphocyte-related subcluster SELENOP-Mφ characterized by increased expressions of *CD3D, FOLR2, IL32*, and *LTC4S*, which might play an antitumor role in LUAD, SELENOP-Mφ is involved in the progression of LUAD via regulating peptide metabolism, protein transport and cytokine secretion. Considering the impact that immunotherapy might have on NSCLC, this study is essential since it allows us to understand how different immune responses can be explained by investigating and identifying subtype differences in the immune microenvironment of these patients (76). A four-gene prognostic signature was identified through scRNA-seq in a TCGA-LUAD cohort that can be independently used to predict outcomes (HR = 1.925; 95% CI: 1.405-2.63) and immune landscapes for LUAD patients. Furthermore, patients could be stratified considering three different immune patterns based on macrophage-related genes (149). A predictive model consisting of nine hub genes associated with the progression of LUAD and correlated with macrophages, B cells, CD8 + T cells, neutrophils, CD4 + T cells and dendritic cells was proposed by Zhong et al. (2021) to be correlated with immune infiltration and providing new potential clinical strategies for LUAD patients (150). While assessing the immune landscape and its role in the initiation and development of LUAD, another study focused on tumor-associated macrophages, it was observed that VEGFA, TIMP1 and SPP1 are critical regulatory molecules of macrophages in LUAD, being overexpressed in immunosuppression-associated macrophages and significantly associated with patient’s survival. Moreover, an alteration in macrophage functions and a connection between the infiltration of macrophages in the immune microenvironment and tumorigenesis have been observed (151). Wang et al. demonstrated by integrating scRNA-seq and bulk RNA sequencing data that CD36 participates in regulating the differentiation of macrophages, and high expression of CD36 is correlated with poor prognosis of LUSC patients. Also, the study has performed a screening from the CTD database of small molecular compounds (e.g. estradiol, alitretinoin, dexamethasone, retinol acetate and cholesterol) that can decrease the CD36 expression. Their observations indicated these small molecules efficiently reduce CD36 expression, demonstrating their potential as a targeted treatment for LUSC (136). Hence, we can conclude that scRNA-seq technology is applied to make observations regarding some of the key endeavors (82) related to drug discovery – target identification (136, 150, 151), preclinical model selection (147, 148), and drug response and disease monitoring.

## Challenges

While providing a wealth of information, scRNA-seq also comes with several challenges, which will be only briefly mentioned here (for a more detailed review, see (152–154)). The most obvious challenge is represented by the high costs of both sample preparation and machines, which limit the number of samples profiled in many studies, often requiring the integration of the bulk RNA-seq data available in the public databases to deepen our insights in tumor biology (155). The existence of different sequencing platforms impacts cell assessment in scRNA-seq, as shortcomings may appear due to inefficient cell capture, different protocols may fail to generate a satisfactory number of reads for protein-coding genes, thus manipulating the generation of accurate gene expression profiles which affects reproducibility across studies (45). Although plenty of studies assess biomarkers with prognostic/diagnostic significance (as outlined above), most of these models are constructed using data from public datasets (111, 156) and still need to be validated in clinical studies. Since scRNA-seq is susceptible to sample quality, poor handling and suboptimal preservation often lead to confounding results (155). An alternative to using fresh tissue samples is using FFPE samples, which can be stored long-term, thus enabling large scale retrospective studies, although RNA degradation and fragmentation in FFPE samples can hinder data quality and interpretation (157). There are many studies assessing intratumoral heterogeneity dealing with shortcomings regardingthe archived tissue samples as there are challenges to accurately compare cellular populations derived from different tumour sites, circulating tumour cells, and patient-derived xenografts (132). Although scRNA-seq is a comprehensive technology, no information about protein abundance or post-translational modifications are generated, indicating that an exhaustive characterization of single cells should also include proteomics and epigenomics (ATAC-seq) analyses (158). Moreover, tumor heterogeneity characterization, especially in the TME is affected by the loss of spatial information caused by tissue dissociation, thus underlining the necessity of combining scRNA-seq with spatial transcriptomics technology to assess tumor heterogeneity and tumor infiltration levels, to offer important information, particularly for drug discovery in cancer (159). In addition, this technique allows only a limited number of cells to have their transcriptomic profile measured, and in these circumstances, lowly expressed genes may remain overlooked (155). Another restraint is encountered in rare malignancies, where there is only incomplete or insufficient reference data to annotate all cell types identified (160). In longitudinal studies, where therapy efficiency or resistance mechanisms are evaluated, clinically actionable conclusions are difficult to obtain, among other reasons, because paired samples are not always collected from the same patient, and insufficient clinical data are available for these patients (161). Finally, different ways of analysing data often lead to somewhat different results, partly due to the inconsistent implementation of algorithms in other software packages (162). Ultimately, analyzing data is only a starting point for a testable hypothesis, and experiments in a relevant biological system must follow up.

## Conclusion and future developments

In this work, we illustrated the use of scRNA-seq techniques in translational research, particularly in lung cancer. This holds great potential for both mechanistic and fundamental insights and practical applications to disease understanding and drug discovery. Some challenges concerning sample acquisition, data analysis, interpretation, and actionability remain, which we have briefly summarized and will hopefully be alleviated with further developments.

Improving the transparency and reproducibility of scRNA-seq studies starts with making the data truly usable for others—sharing the raw files, the processed matrices, and clear explanations of what was done at each step. Providing the analysis code, rather than only the final figures, helps others understand exactly how the results came together. Using established analysis pipelines can prevent avoidable errors, and being open about why certain filters or parameters were chosen makes the workflow easier to follow. Cell-type labels should be supported by straightforward marker evidence instead of relying entirely on automated tools. Just as importantly, the main conclusions should be tested in independent datasets to make sure they are not specific to a single experiment, especially given how much patient-to-patient variation exists. When data are easy to access, methods are easy to understand, and results can be checked elsewhere, single-cell studies become far more reliable and far more useful for researchers trying to turn these insights into real clinical advances.Patient diversity has a meaningful impact on what single-cell studies reveal, as tumors are widely different. Ethnicity, environmental exposures, as well as lung cancer subtype can all influence tumor cells behavior within the tumor. Future studies should deliberately include a broader range of patients and clearly describe groups represented in the analysis. By acknowledging and reporting diversity in this way, single-cell studies become more trustworthy, more inclusive, and better aligned with the real variability seen in clinical populations.
